# Comparison of multi‐institutional Varian ProBeam pencil beam scanning proton beam commissioning data

**DOI:** 10.1002/acm2.12078

**Published:** 2017-04-19

**Authors:** Ulrich W. Langner, John G. Eley, Lei Dong, Katja Langen

**Affiliations:** ^1^ Department of Radiation Oncology Maryland Proton Treatment Center University of Maryland School of Medicine 850 W. Baltimore Street Baltimore MD 21201 USA; ^2^ Scripps Proton Therapy Center 9730 Summers Ridge Road San Diego CA 92121 USA

**Keywords:** Probeam, Proton, spot scanning

## Abstract

**Purpose:**

Commissioning beam data for proton spot scanning beams are compared for the first two Varian ProBeam sites in the United States, at the Maryland Proton Treatment Center (MPTC) and Scripps Proton Therapy Center (SPTC). In addition, the extent to which beams can be matched between gantry rooms at MPTC is investigated.

**Method:**

Beam data for the two sites were acquired with independent dosimetry systems and compared. Integrated depth dose curves (IDDs) were acquired with Bragg peak ion chambers in a 3D water tank for pencil beams at both sites. Spot profiles were acquired at different distances from the isocenter at a gantry angle of 0° as well as a function of gantry angles. Absolute dose calibration was compared between SPTC and the gantries at MPTC. Dosimetric verification of test plans, output as a function of gantry angle, monitor unit (MU) linearity, end effects, dose rate dependence, and plan reproducibility were compared for different gantries at MPTC.

**Results:**

The IDDs for the two sites were similar, except in the plateau region, where the SPTC data were on average 4.5% higher for lower energies. This increase in the plateau region decreased as energy increased, with no marked difference for energies higher than 180 MeV. Range in water coincided for all energies within 0.5 mm. The sigmas of the spot profiles in air were within 10% agreement at isocenter. This difference increased as detector distance from the isocenter increased. Absolute doses for the gantries measured at both sites were within 1% agreement. Test plans, output as function of gantry angle, MU linearity, end effects, dose rate dependence, and plan reproducibility were all within tolerances given by TG142.

**Conclusion:**

Beam data for the two sites and between different gantry rooms were well matched.

## 1 Introduction

Proton pencil beam spot scanning is an emerging technology that is increasingly used in proton centers around the world. Spot scanning provides the ability to modulate the beam in energy as well as intensity as the dose is painted across the target. Spot scanning also negates the use of collimators and compensators, which are sources of neutron dose to the patient. Varian Medical Systems is among the latest vendors entering the proton market with their ProBeam™ spot scanning system. Two facilities in the United States currently use the ProBeam system, and several more are in different phases of construction and commissioning in the United States and Europe.^1^ The first center using the Varian ProBeam system in the United States was the Scripps Proton Therapy Center (SPTC, San Diego, CA, USA). The Maryland Proton Treatment Center (MPTC) (Baltimore) was the second. With two centers and three gantries at each currently operational, we compared commissioning data to determine how well different sites can be matched. Commissioning of a spot scanning system with a synchrotron was described previously,^2^ but not for a Varian ProBeam system with a cyclotron and not comparing commissioning data across sites. ProBeam systems are exclusively spot scanning systems, with no passive scattering components. Monitor unit (MU) linearity, end effects, dose rate dependence, and reproducibility for the system at MPTC are also discussed here. This study describes dosimetric commissioning tests used for the Varian ProBeam system at MPTC and SPTC and provides results that can be used as a reference for future ProBeam sites. Although the tests described here are not complete, they are similar to those used before^2^, ^3^, ^4^ and should provide useful benchmarks.

## 2 Methods and materials

The treatment planning system (TPS) used by both sites is the Varian Eclipse v11, and the treatment machine software version is ProBeam v2.7. To commission the pencil beam proton convolution superposition dose model for the TPS the integrated depth dose curves (IDDs), absolute dose calibration, and spot profiles in air had to be measured, as outlined by the Eclipse reference manual. A complete description of the dose model is given in the Eclipse V11 Proton Algorithm Reference Guide.^5^ These measurements were compared for the two facilities. The TPSs were then verified by dosimetric validation of various test plans, similar to what is suggested in reports from the American Association of Physicists in Medicine and others.^6^, ^7^, ^8^ Data from the first clinical gantry were used to commission the TPS beam model at MPTC. All subsequent gantries at MPTC were compared to the initial data to determine dosimetric equivalence.

### 2.A Beamline

The Varian ProBeam system exclusively uses spot scanning gantries that dynamically scan the beam from one spot to another (if distances between spots are less than a few millimeters). The system uses a superconducting isochronous cyclotron with an azimuthally varying field to accelerate hydrogen nuclei. This technology allows proton acceleration to 250 MeV with a maximum of 800‐nA extracted current at the exit of the cyclotron. The energy is then modulated continuously (as opposed to a synchrotron system, where the energy is changed discretely) by a double‐carbon wedge degrader system, which can reduce the energy continuously to 70 MeV. Typically a current of 1–2 nA is used during patient treatment, but nozzle current can be as high as 10 nA. Beam losses in the energy selection system can range from 98% (250 MeV) to 99.75% (70 MeV).^9^ The beam nozzle contains two steering magnets for the beam, a kapton window to seal the vacuum, an MU chamber, and a strip ionization chamber to verify the beam position. The center of the *y*‐steering magnet is at 256 cm and the *x*‐steering magnet at 200 cm. A source‐to‐isocenter distance of 228 cm is thus used in the TPS. The maximum field size is 30 (*x*) by 40 (*y*) cm at isocenter, where the *y* axis is aligned in the craniocaudal direction in a Head first supine (HFS) patient with the table at the nominal treatment position. A range shifter can be inserted into the snout, and the snout can move continuously from 3 to 42 cm from the isocenter. The gantry can rotate 360°, and the couch can rotate from 265° to 95°, with 0° as the nominal position. For pitch and yaw 3° are allowed clinically. The planned energy layer switch time is <1 s, and the minimum time to deliver the minimum weighted spot per energy layer is ~3 ms. The spot with the smallest number of MUs needed per layer will thus determine the dose rate. The smallest sigmas of the spots are ~4 mm in air at isocenter.

The MPTC has four gantry rooms (TR1, TR2, TR3, and TR4) and one fixed‐beam room. SPTC has three gantry rooms and two fixed‐beam rooms. SPTC data used here for comparison reflect average data for all their gantries after commissioning.

### 2.B Measuring Bragg peaks

The IDDs were acquired with a PTW Bragg peak chamber (PTW‐Freiburg, Germany) in a 3D water tank at both sites. The Bluephantom2 (IBA Dosimetry, Schwarzenbruck, Germany) was used at MPTC and the PTW 3D water tank (PTW‐Freiburg, Germany) at SPTC. The measuring field diameter of the Bragg peak chamber is 8.4 cm, and the active volume is 10.5 cm^3^. The window water equivalent thickness (WET) is 4.0 mm. IDDs were also acquired with a Stingray ion chamber (IBA Dosimetry). The measuring field diameter of this chamber is 12 cm and the window WET is 4.9 mm. The maximum energy that can be delivered in the room is 245 MeV, with a range of ~38.5 cm. Bragg peak range measurements were compared to the theoretical calculation by applying the Bortfeld equation (R_80_ = 0.00244*E^1.75^),^10^, ^11^ where E represents the energy and R_80_ the depth of the distal 80% of the maximum dose value of the Bragg peak in water. Although the Bortfeld equation is an approximation, it gives a good measure of expected theoretical values for clinically used energies. Bragg peaks were acquired from a gantry angle of 90° in an International Electrotechnical Commission (IEC) 61217 coordinate system, because the water tank was not deep enough to acquire the Bragg peaks from a gantry angle of 0° for the highest energies. Lower energy IDDs were verified with measurements from a gantry angle of 0°. Single pencil beam scans at isocenter were consequently acquired from the side of the water tank, where a 5‐mm‐thick 20 × 20 cm^2^ window was inserted with a WET of 5.5 mm. These scans were acquired every 10 MeV from 70 to 245 MeV. In order to acquire the Bragg peaks in the surface region, the first 10 cm of each IDD was acquired at a gantry angle of 0° and then normalized and combined with deeper IDD data. A PTW 7862 chamber was used as reference chamber. The diameter of this chamber is 9.65 cm, with a physical window thickness of 0.2 mm. The measured IDDs were corrected for the WETs of all the material between the reference chamber surface and the inside surface of the Bragg peak chamber.

Bragg peaks were also verified and compared using a Giraffe multilayer ion chamber (MLIC) device (IBA Dosimetry) that contains 180 air‐vented parallel‐plate ion chambers with diameters of 12 cm. The chambers are spaced 2 mm apart. The Giraffe was also used to verify the WETs of the reference chamber, the range shifters, and the water tank window.

### 2.C Absolute calibration

The absolute output of the unit was measured using the methodology recommended by the TRS 398 report of the International Atomic Energy Agency^12^ for determination of absorbed dose from a proton beam. A PPC05 Markus parallel‐plate chamber (IBA Dosimetry) was used in a 10 × 10 cm^2^ field of mono‐energetic spots spaced 2.5 mm apart, resulting in 1,681 spots per layer with 10 MU delivered per spot. Each energy was measured separately in intervals of 10 MeV (corresponding to the measured IDDs). The window for this chamber has a physical thickness of 1 mm and a WET of 1.8 mm. A point with 2‐cm water equivalent depth was then used as the absolute measurement point for all the energies at MPTC and 1.5 cm at SPTC. All data were renormalized to 2 cm for comparison. The water tank was moved to place the isocenter at the effective point of measurement of the chamber to eliminate the need for a source‐to‐axis‐distance correction, and a gantry angle of 0° was used for these measurements. The corresponding IDDs at MPTC for each energy were then scaled according to this measurement at a 2‐cm depth for import into the planning system in units of Gy.mm^2^/MeV.

Relative biological effectiveness (RBE) was chosen as 1.1 and was incorporated in our planning system through a depth–dose normalization table. The planning system (Eclipse) consequently provides dose in cGy RBE. Incorporating the RBE through the table and not through scaling the IDDs makes it easier to adjust the RBE in future if necessary.

### 2.D Spot profiles

Spot profiles were measured with the Lynx device (IBA Dosimetry), which uses a scintillator screen to record proton interactions and has a resolution of 0.5 mm. Monoenergetic spots were delivered on the central axes in 10‐MeV intervals. Measurements of spot profiles in air were required for the TPS at the isocenter and 10 and 20 cm above and below to calculate the effective source position and divergence of the beam. With the range shifters inserted, the snout position was placed at 26 cm to more closely resemble most clinical treatment scenarios. Spot profiles were also measured as functions of gantry angle every 30° using a couch‐mounted device. Analysis of the profiles was then performed with in‐house code (Matlab, Mathworks, Natick, MA, USA) to determine the full width at half maximum (FWHM) and sigma (*σ*) of the spots for each energy on both in‐plane axes from the measured intensities. Sigma is calculated from the FWHM of the spot profile for both *x* and *y* axes.

The sigmas were then evaluated for spot size as a function of energy and gantry angle as well as symmetry between the *x* and *y* profiles.

Symmetry between the *x* and *y* profiles of each spot was defined here as |(σ_x_‐σ_Y_)|/(σ_x_+σ_Y_)*100% and calculated for each spot.

### 2.E MU linearity (tolerance: ≤2% for >5 MU and ≤5% for ≤5 MU)

For MU linearity, monitor end effect, and dose rate dependence, a PPC05 parallel‐plate ion chamber was used in water. The chamber's effective point of measurement was placed at a 2‐cm depth. Dose rates used for linearity measurements were 60,000 MU/min for 70 MeV, 500,000 MU/min for 160 MeV and 200,000 MU/min for 240 MeV for MU settings of 2, 5, 50, 100, 500, 1,000, 2,500, 5,000, and 10,000 MU, for a monoenergetic pencil beam on the central axis. Readings were normalized to the 100‐MU reading for each energy.

### 2.F Monitor end effect (tolerance: ≤3 MU for 3,000 MU deliveries [0.1%])

The dose rate used was 60,000 MU/min, and the end effect was measured for 70, 160, and 240 MeV for a complete delivery of 3,000 MU and a delivery of 3,000 MU in three separate 1,000‐MU deliveries. The end effect was calculated by assuming that ionization M in general is proportional to the sum of *n* times the set number of monitor units and *n* times the end effect (T_E_), expressed as:

(1) $$M=n\left(MU+T_{E}\right),\;i.e.\;TE=\left(M_{3}-M_{1}\right)/\left(3^{*}M_{1}-M_{3}\right)\times T,$$

where T is the total number of MUs, M_1_ is the measurement with no interruptions, and M_3_ the measurement with *n* = 3 interruptions.

The end effect was also calculated by fitting a linear regression through the data measured for the linearity.

### 2.G Dose rate dependence (tolerance: ≤2%)

A fixed dose of 2,000 MU was delivered with a monoenergetic pencil beam on the central axis for 70 and 160 MeV and 500 MU for 240 MeV. Different dose rates were used for each energy (i.e., 6,500, 50,000, 100,000, 1,000,000, 5,000,000 MU/min for 70 MeV; 50,000, 100,000, 750,000, 1,500,000, 3,000,000 MU/min for 160 MeV; and 10,000, 20,000, 40,000, 80,000, 250,000, and 500,000 MU/min for 240 MeV). Values were normalized to the maximum delivered dose rate for each energy. The number of MUs had to be decreased for higher energies to reduce the current of the proton beam at isocenter. These dose rates cover the clinically expected dose rates.

### 2.H Output vs gantry angle (tolerance: ≤1%)

A small‐volume CC04 ion chamber (IBA Dosimetry) was used inside a 5‐cm WET buildup cap attached to the edge of the couch. The gantry was rotated to the four cardinal gantry angles. Plans with spots covering a 10 × 10‐cm^2^ monoenergetic plane were delivered for 70, 160, and 240 MeV. Spot spacing in these plans was 2.5 mm, and each spot was equally weighted. Values were normalized to the measurements at gantry = 0°. A dose rate of 60,000 MU/min was used for all energies, and 10,000 MUs were delivered at each angle.

### 2.I Reproducibility and interrupted treatment (tolerance: Less than larger of 0.5 cGy or 1% of delivered dose)

The gantry was placed at 0°, and the Matrixx PT (IBA dosimetry) planar ion chamber array was placed at the isocenter with a 5‐cm buildup (5.4‐cm WET) added to place the measuring point at 6 cm. Three plans were delivered with different ranges and spread‐out Bragg peaks (SOBPs) to cover a wide range of energies. Each plan covered a 26 × 26‐cm^2^ surface to a dose of 500 cGy for varying SOBPs (i.e., R8S4, R15S7, and R22S7, where R represents the nominal range in centimeters and S the length of the SOBP in centimeters). Each plan was delivered three times, and doses at the central chamber were recorded and compared.

To test an interrupted treatment, arbitrary fields were repeatedly delivered on the Matrixx PT array. For the first delivery, the complete field was delivered uninterrupted. For the second field, treatment was interrupted at approximately halfway and then restarted. Profiles for these measurements were compared and analyzed.

### 2.J Test plans

Verification plans were run at MPTC to verify the TPS. Plans were calculated with the commissioned TPS for 36 10 × 10 × S cm^3^ volumes, where S represents different SOBPs in a 40 × 40 × 40 cm^3^ water phantom created in the TPS with the isocenter at 20‐cm depth in water on the central axis. Nine plans were for open fields, and nine for each range shifter. Plans for S = 2, 3, and 10 cm were used with nominal energies of 140, 200, and 230 MeV. Sixteen plans were also used for 5 × 5 × 5 cm^3^ of axis volumes, four for open fields, and four for each range shifter. In addition, a 15 × 15 × 15 cm^3^ and a 20 × 20 × 20 cm^3^ plan were evaluated. Resulting calculations were then compared with measurements acquired with the PPC05 parallel‐plate chamber in the 3D water tank. Another set of test plans was also created in Eclipse to simulate various clinical treatment scenarios (e.g., targets at different depths, different target widths and thicknesses, off‐axis targets, different range shifters, etc.). The gantry was at 0°, and all fields were anterior–posterior beams.

### 2.K Range shifters and couch base

Three range shifters, with physical thicknesses of 5, 3, and 2 cm were commissioned for each gantry. The WETs of the range shifters, reference ion chamber, water tank window, and kVue One Proton couch base (Qfix, Avondale, PA, USA) were verified with the Giraffe MLIC and compared to the theoretical values provided by Varian for the acrylic material of the range shifters.

## 3 Results and discussion

### 3.A Bragg peaks

Data in Table 1 show that differences in R_80_ between the theoretical and measured values are <1 mm from 90 to 200 MeV (tolerances used: <=0.5 mm for <75 MeV and <=1 mm for 80–210 MeV). For 80–210 MeV the difference in R_80_ is a maximum of 1.5 mm between the MPTC gantries and the theoretical values, which is larger than the tolerance. This difference between the MPTC gantries and the theoretical values increases from 2.5 mm at 220 MeV to a maximum of 4.4 mm at 240 MeV. These values were within tolerance from the values given for acceptance testing by the vendor, which were calculated with Monte Carlo modeling incorporating the energy spread in the beam. The theoretical values assume no energy spread introduced by the beam line and the larger difference is thus acceptable and in accordance with the values given by the vendor.

**Table 1:** Data measured with the IBA 3D water tank and a PTW 34070 ion chamber (TR4 and TR3) and a Stingray ion chamber (TR1), comparing TR1 with TR4 and TR3 with TR4. Theoretical values for each energy were calculated from the Bortfeld equation (R_80_ = 0.00244×E^1.75^)

Requested energy (MeV)	Theoretical values (cm)	TR4 R80 measured (cm)	TR3 R80 measured (cm)	TR1 R80 measured (cm)	TR4 Energy calculated from measured R_80_ (MeV)	TR3 Energy calculated from measured R_80_ (MeV)	TR1 Energy calculated from measured R_80_ (MeV)	%diff R_80_ (%) (TR1 from TR4)	%diff R_80_ (%) (TR3 from TR4)	%diff Energy (%) (TR1 from TR4)	%diff Energy (%) (TR3 from TR4)
80	5.22	5.17	5.09	5.08	79.55	78.84	78.75	−1.741	−1.547	−0.998	−0.887
90	6.42	6.39	6.37	6.33	89.79	89.62	89.30	−0.939	−0.313	−0.538	−0.179
100	7.72	7.71	7.69	7.65	99.96	99.81	99.51	−0.778	−0.259	−0.445	−0.148
110	9.12	9.12	9.10	9.07	110.02	109.89	109.68	−0.548	−0.219	−0.314	−0.125
120	10.62	10.68	10.55	10.61	120.41	119.57	119.96	−0.655	−1.217	−0.375	−0.697
130	12.21	12.25	12.15	12.19	130.23	129.62	129.87	−0.490	−0.816	−0.280	−0.467
140	13.90	13.94	13.88	13.91	140.21	139.87	140.04	−0.215	−0.430	−0.123	−0.246
150	15.69	15.73	15.69	15.68	150.23	150.01	149.96	−0.318	−0.254	−0.182	−0.145
160	17.56	17.60	17.57	17.56	160.19	160.04	159.98	−0.227	−0.170	−0.130	−0.097
170	19.53	19.56	19.56	19.53	170.16	170.16	170.01	−0.153	0.000	−0.088	0.000
180	21.58	21.58	21.58	21.56	179.98	179.98	179.89	−0.093	0.000	−0.053	0.000
190	23.73	23.71	23.68	23.67	189.93	189.79	189.75	−0.169	−0.127	−0.096	−0.072
200	25.95	25.89	25.86	25.86	199.72	199.59	199.59	−0.116	−0.116	−0.066	−0.066
210	28.27	28.17	28.16	28.12	209.59	209.55	209.38	−0.177	−0.035	−0.101	−0.020
220	30.66	30.48	30.48	30.41	219.24	219.24	218.96	−0.230	0.000	−0.131	0.000
230	33.14	32.90	32.87	32.86	229.03	228.91	228.87	−0.122	−0.091	−0.069	−0.052
240	35.71	35.30	35.32	35.31	238.43	238.51	238.47	0.028	0.057	0.016	0.032

Table 1 also shows a difference in the delivered and calculated beam energies of <1.0 MeV for energies between 70 MeV and 210 MeV for the MPTC gantries. This difference increases to a maximum of 1.6 MeV for the MPTC gantries at 240 MeV. The maximum percentage difference of 1.6% between the requested and calculated energies is, however, at 70–80 MeV, whereas for energies >80 MeV the percentage differences were all <0.7%. Comparisons of TR1 and TR3 percentage differences in energy and R_80_, with that of TR4, show that all differences were <1% for both for energies >80 MeV. For 80 MeV the differences in R_80_ were the largest at 1.74% for TR1, compared to TR4, and 1.55% for TR3, respectively, corresponding to 0.9 and 0.8 mm differences in range in water.

**Figure 1:**
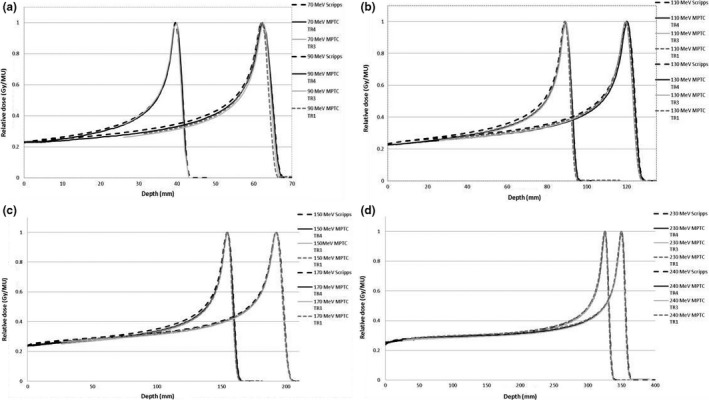
Relative integrated depth dose as function of depth as measured for MPTC gantries TR1, TR3, and TR4 and SPTC at energies of 70, 90, 110, 130, 150, 170, 230, 240 MeV.

IDDs for the MPTC gantries were also compared with those from SPTC. The data were in good agreement (Fig. 1) for all energies in the Bragg peak region and for energies >170 MeV in the plateau and shoulder regions. For energies <170 MeV the SPTC IDDs were higher than the MPTC IDDs, especially in the shoulder region. The IDDs for TR3 and TR4 were measured with the 8.4‐cm Bragg peak chamber (because the Stingray was not available at that time), and the IDDs for TR1 were measured with both the Stingray and PTW Bragg peak chambers. This was done to validate measurements of the PTW Bragg peak chamber with the larger volume Stingray Bragg peak chamber. The IDDs for these gantries and chambers are similar. However, for higher energies the IDDs in the shoulder region for TR1 (larger chamber) are slightly higher than those for TR4 (smaller chamber), suggesting that the chamber is not large enough for these higher energies to capture all secondary protons in the halo. This agrees with data from Monte Carlo studies on the halo effect.^13^, ^14^, ^15^, ^16^ The increase in dose of the SPTC data was on average 4.5% higher for lower energies compared to that of MPTC. SPTC introduced Monte Carlo–modeled data into their data which take the halo effect more accurately into account. However, this difference occurs in the plateau region, which is only ~25% of the maximum dose (i.e., the effect is more on the order of ~1% if this region contributes dose to the target volume). We observed no marked differences in comparisons of the TPS plans with measured data inside the planning target volume. This increase in the plateau region decreased as energy increased, and there was no marked difference for energies >180 MeV.

**Figure 2:**
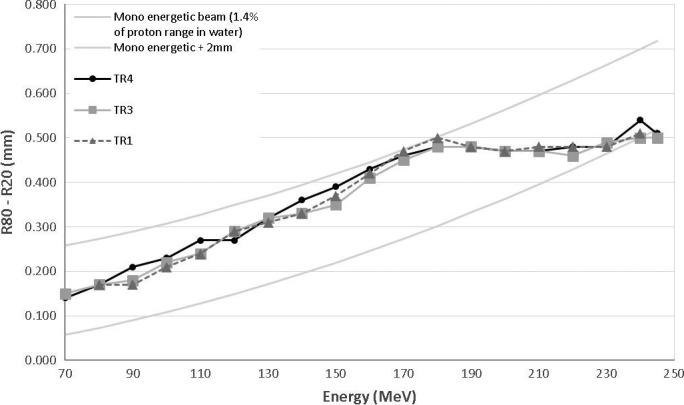
Distal penumbra of the Bragg peaks (R_80_–R_20_) for MPTC gantries TR1, TR3, and TR4 as measured in a water tank with PTW and Stingray Bragg peak chambers. Tolerances represent values <0.2 g/cm^2^ above the physical limit from range straggling of a monoenergetic beam (1.4% of the proton range in water) for each energy and are indicated by solid lines.

Distal penumbra between R_80_ and R_20_ in the distal edge of the Bragg peak measured with the Giraffe MLIC are shown in Table 2 for each of the MPTC gantries. These values were also acquired with the water tank and the Bragg peak chamber and are shown in Fig. 2. The tolerance used was: <0.2 g/cm^2^ above the physical limit from range straggling of a monoenergetic beam (1.4% of the proton range in water). All measured values were smaller than the tolerances for each energy. The differences between the values measured for TR1 and TR3 compared to TR4 were all <0.03 cm.

**Table 2:** Distal fall‐off (R80–R20) measured for MPTC gantries TR1, TR3, and TR4 with the IBA Giraffe multilayer ion chamber array. Tolerance is <0.2 g/cm^2^ above the physical limit from range straggling of a monoenergetic beam (1.4% of the proton range in water). Water tank data are shown in Fig. 2

Requested energy (MeV)	TR1			TR3			TR4		
	R80 measured (cm)	R20 measured (cm)	Distal fall‐off (R80–R20) (cm)	R80 measured (cm)	R20 measured (cm)	Distal fall‐off (R80–R20) (cm)	R80 measured (cm)	R20 measured (cm)	Distal fall‐off (R80–R20) (cm)
70	4.05	4.19	0.14	–	–	–	4.04	4.18	0.14
80	5.14	5.32	0.18	5.08	5.25	0.17	5.16	5.34	0.18
90	6.39	6.57	0.18	6.36	6.60	0.24	6.39	6.60	0.21
100	7.71	7.93	0.22	7.67	7.89	0.22	7.70	7.94	0.24
110	9.14	9.39	0.25	9.07	9.33	0.26	9.11	9.38	0.27
120	10.66	10.96	0.30	10.53	10.82	0.29	10.63	10.93	0.30
130	12.26	12.59	0.33	12.12	12.46	0.34	12.23	12.56	0.33
140	13.96	14.31	0.35	13.87	14.21	0.34	13.93	14.30	0.37
150	15.74	16.12	0.38	15.65	16.03	0.38	15.71	16.12	0.41
160	17.61	18.06	0.45	17.54	17.98	0.44	17.57	18.03	0.46
170	19.62	20.11	0.49	19.55	20.04	0.49	19.56	20.05	0.49
180	21.65	22.17	0.52	21.58	22.09	0.51	21.58	22.09	0.51
190	23.76	24.29	0.53	23.69	24.21	0.52	23.70	24.23	0.53
200	25.96	26.48	0.52	25.88	26.39	0.51	25.89	26.41	0.52
210	28.21	28.74	0.53	28.18	28.70	0.52	28.16	28.70	0.54
220	30.57	31.11	0.54	30.54	31.07	0.53	30.53	31.08	0.55
230	32.92	33.43	0.51	32.87	33.37	0.50	–	–	–

The sharpest distal fall‐off occurred for the lower energies because of less range straggling. The effect of the carbon energy degrader is evident from the almost constant slope for energies >180 MeV. This was first described by Hsi et al.^17^ and is only evident in systems with an energy degrader and energy selection slit. This is caused by the increased spread in the energy spectrum for lower energies caused by the degrader. If the full‐energy spread generated in the degrader is transported to the treatment location, the width of the peak measured in water should be constant as a function of range. The introduction of the energy slit to reduce the spread in energy causes this continued decrease as the range decrease below 180 MeV. The width of the slit will thus determine where this transition from a constant to a decreasing slope will occur and is fixed for all Varian ProBeam systems. Above 180 MeV the full‐energy spectrum is transported but not below that, causing range straggling to become dominant.

### 3.B Absolute calibration

**Figure 3:**
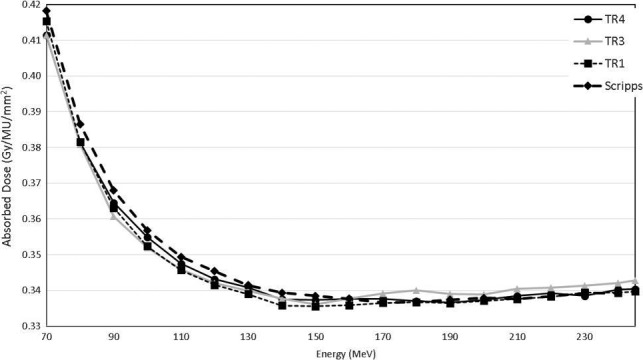
Absolute calibration measurements as functions of energy according to the TR398 protocol for MPTC gantries TR1, TR3, and TR4 measured at a depth of 2 cm for a 10 × 10 cm^2^ monoenergetic field. The isocenter was placed at the same depth as measurement. SPTC measurements were normalized with those of TR4 at 160 MeV.

Measured absolute doses as functions of energy are shown in Fig. 3 for each gantry as well as for SPTC. The SPTC doses were normalized at 160 MeV to that of the TR4 to eliminate any discrepancies between MPTC calibration and that of SPTC. The absolute doses for the gantries at MPTC and SPTC are all within 1% of those measured for TR4. The doses of TR4 were used in the TPS. After commissioning of the Eclipse treatment planning system, doses were recalculated for the same setup used during measurement. The TPS calculated doses were then compared with the measured doses to quantify discrepancies between the TPS and measurements. Measured values of the MPTC gantries were all within 1.5% from those of the TPS, except for 245 MeV, which was at 2.7% for all gantries. Calibration of the monitor ion chamber in the snout had to be adjusted for TR3 to achieve better agreement with TR4 values.

The decrease in the output as a function of energy and the eventual slight increase for energies >140 MeV can be attributed to the energy slit, which is used to keep energy dispersion low after the degrader, similar to earlier descriptions.^17^

### 3.C Spot profiles

#### 3.C.1 Spot size (Tolerance: |*σ*| ≤15%)

**Figure 4:**
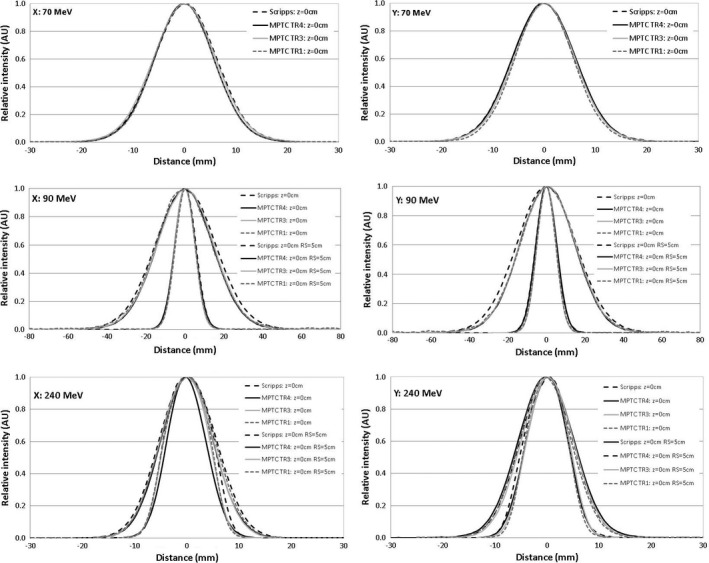
Comparison of spot profiles for MPTC gantries TR1, TR3, and TR4 and SPTC for 70, 90, and 240 MeV without a range shifter and 90 and 240 MeV with a 5‐cm range shifter. The snout position with the range shifter was 26 cm. These profiles were measured in air at the isocenter. Larger FWHM plots represents the spot profiles with the 5‐cm range shifter.

Spot profiles measured for each gantry at isocenter and selected energies (70, 90, and 240 MeV) without a range shifter are shown in Fig. 4. The FWHM of the profiles is slightly larger for SPTC and TR1 than TR4 at higher energies and smaller at lower energies, but within the 15% tolerance compared to each other. Profiles with the 5‐cm range shifter inserted also agree well. In Table 3 calculated *σ*s are shown in 10‐MeV steps for the MPTC gantries. Root mean squares (RMSs) were computed over all the MPTC gantries for all *σ*s at each measured energy. The *σ*_*x*_ and *σ*_*y*_ values for each gantry were then compared to the RMSs to calculate the RMS error (RMSE) of each *σ* at each energy. The largest average RMSEs over all energies were for TR4 at 0.176 mm. The maximum error occurred for TR3 and was 0.538 mm at 90 MeV. RMSEs were larger for all the gantries at energies >200 MeV, with the exception of 90 MeV for TR1 and TR3. The beam optic model was refined for TR1 and TR3 from that of TR4 at ~180 MeV to achieve a more continuous optical solution. The optical solution also changes for energies >180 MeV, which explains the larger RMSE.

**Table 3:** *σ*s for each MPTC gantry (TR4, TR3, and TR1) and the root mean square error (RMSE) from the average over all treatment rooms. Values were measured at a gantry angle of 0° at isocenter

Energy (MeV)	RMS All gantries σ_x_ and σ_y_	TR4				TR3				TR1			
		σ_x_ (mm)	RMSE (mm)	σ_y_ (mm)	RMSE (mm)	σ_x_ (mm)	RMSE (mm)	σ_y_ (mm)	RMSE (mm)	σ_x_ (mm)	RMSE (mm)	σ_y_ (mm)	RMSE (mm)
245	4.007	3.61	0.397	3.86	0.147	4.15	0.143	3.99	0.017	4.20	0.193	4.20	0.193
240	4.114	3.71	0.404	3.93	0.184	4.26	0.146	4.02	0.094	4.36	0.246	4.36	0.246
230	4.101	3.70	0.401	3.76	0.341	4.23	0.129	4.08	0.021	4.39	0.289	4.39	0.289
220	4.060	3.67	0.390	3.55	0.510	4.11	0.050	4.15	0.090	4.40	0.340	4.40	0.340
210	4.112	3.93	0.182	3.68	0.432	4.10	0.012	4.16	0.048	4.38	0.268	4.38	0.268
200	4.127	4.00	0.127	3.78	0.347	4.05	0.077	4.16	0.033	4.37	0.243	4.37	0.243
190	4.176	4.12	0.056	3.90	0.276	4.08	0.096	4.22	0.044	4.36	0.184	4.36	0.184
180	4.255	4.19	0.065	4.03	0.225	4.32	0.065	4.28	0.025	4.35	0.095	4.35	0.095
170	4.281	4.35	0.069	4.14	0.141	4.13	0.151	4.28	0.001	4.39	0.109	4.39	0.109
160	4.363	4.37	0.007	4.27	0.093	4.17	0.193	4.42	0.057	4.47	0.107	4.47	0.107
150	4.470	4.60	0.130	4.40	0.070	4.24	0.230	4.57	0.100	4.50	0.030	4.50	0.030
140	4.480	4.25	0.230	4.48	0.000	4.41	0.070	4.63	0.150	4.55	0.070	4.55	0.070
130	4.599	4.59	0.009	4.60	0.001	4.46	0.139	4.72	0.121	4.61	0.011	4.61	0.011
120	4.744	4.89	0.146	4.78	0.036	4.59	0.154	4.82	0.076	4.69	0.054	4.69	0.054
110	4.859	4.85	0.009	4.91	0.051	4.82	0.039	5.01	0.151	4.78	0.079	4.78	0.079
100	5.022	5.19	0.168	5.19	0.168	4.99	0.032	4.97	0.052	4.89	0.132	4.89	0.132
90	5.442	5.21	0.232	5.40	0.042	5.98	0.538	5.97	0.528	5.00	0.442	5.00	0.442
80	5.544	5.47	0.074	5.64	0.096	5.61	0.066	5.64	0.096	5.45	0.094	5.45	0.094
75	5.760									5.76	0.000	5.76	0.000
70	5.967	5.75	0.217	6.15	0.183	6.06	0.093	5.90	0.067				
Average			0.174		0.176		0.128		0.093		0.157		0.157
Max			0.404		0.510		0.538		0.528		0.442		0.442

**Figure 5:**
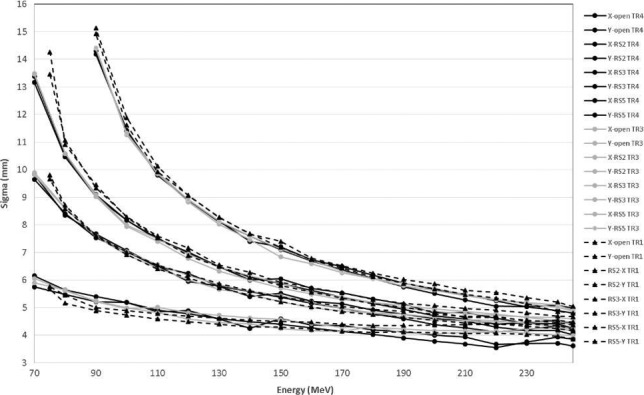
*σ* (x and y directions on the central axis) as a function of beam energy for MPTC gantries for open fields and 2‐, 3‐, and 5‐cm range shifters with a snout position of 26 cm.

In Fig. 5 *σ*_*x*_ and *σ*_*y*_ are shown as functions of energy at a gantry angle of 0° at isocenter for an open field and fields with range shifters of 2, 3, and 5 cm inserted for TR1, TR3, and TR4, respectively. The snout position was at 26 cm from the isocenter for measurements with the range shifters. RMSEs for fields with range shifters were smaller than for open fields. The spot size increased by a factor of 3 when the 5‐cm range shifter was used at 90 MeV.

**Figure 6:**
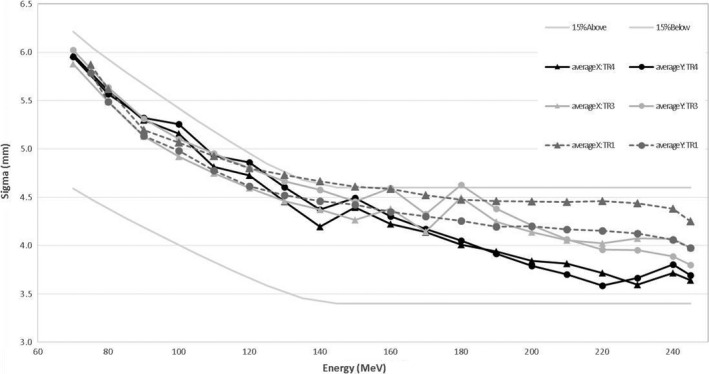
Average *σ* (x and y directions) over gantry angle as a function of beam energy for MPTC gantries. Tolerances from Varian of ±15% are shown in gray.

**Figure 7:**
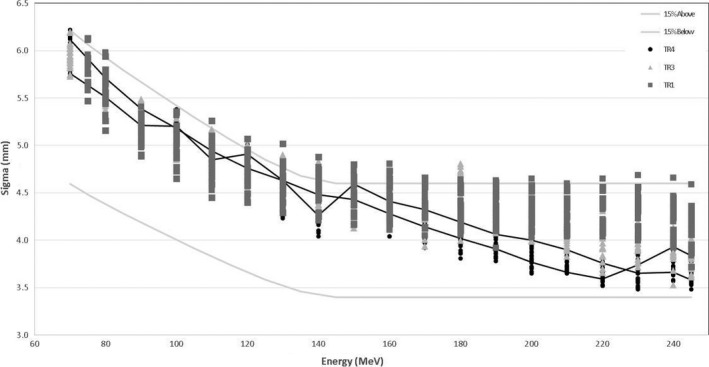
*σ* (x and y directions) as a function of beam energy for MPTC gantries TR1, TR3, and TR4 at 30° gantry angle intervals, starting from 0° to 330°. Tolerances from Varian of ±15% are shown in gray. Values for TR4 at a gantry angle of 0° are shown as a solid black line. These values were used in the treatment planning system model for x and y, respectively.

In Figs. 6 and 7 the distributions of *σs* at isocenter over gantry angle as functions of energy are shown. Profiles were measured at 30° increments starting from gantry angle 0°. Spot profiles measured at a gantry angle of 0° for TR4 were used to commission the TPS, because that was the only gantry available at that time. The other gantries are compared to these values. In Fig. 6 the average values for each gantry over all the gantry angles are shown. In Fig. 7 all the values are shown for each gantry, as well as values for TR4. A gradual increase in *σ* corresponds to relative gantry proximity to the cyclotron (TR4 is the farthest away along the beamline), but no clear link could be found. The tolerances shown (±15%) were given by Varian and found to be acceptable. Differences between beam optics for the gantries are largest at higher energies. The beam optic model was refined for TR1 and TR3 from that of TR4 at ~180 MeV to achieve a more continuous optical solution. Although some values were out of the 15% upper tolerance, especially between 110 and 180 MeV, averages over all gantry angles were within tolerance and the variances of *σ*s were also within 30% and thus acceptable. Variance in spot size over gantry angle can be as large as 20% at the same energy. This is caused by rotation of the gantry as the focused beam coming from the beam line does not rotate. Any asymmetry in beam focus in the *x* plane (relative to the gantry) will thus translate to the *y* plane as the gantry rotates 90°. Differences between gantries are largest at higher energies. Because these profiles were measured in air, these spots will contribute to shallower dose. For higher energies at depth, however, the difference will have a reduced impact, because spot size there depends on elastic scattering and any difference will “smear out.” Impacts on plan quality and equivalence of beam models were evaluated for different *σ*s and were found to have no impact within differences of 20% at high energies (corresponding to ~0.8 mm in *σ*).

**Figure 8:**
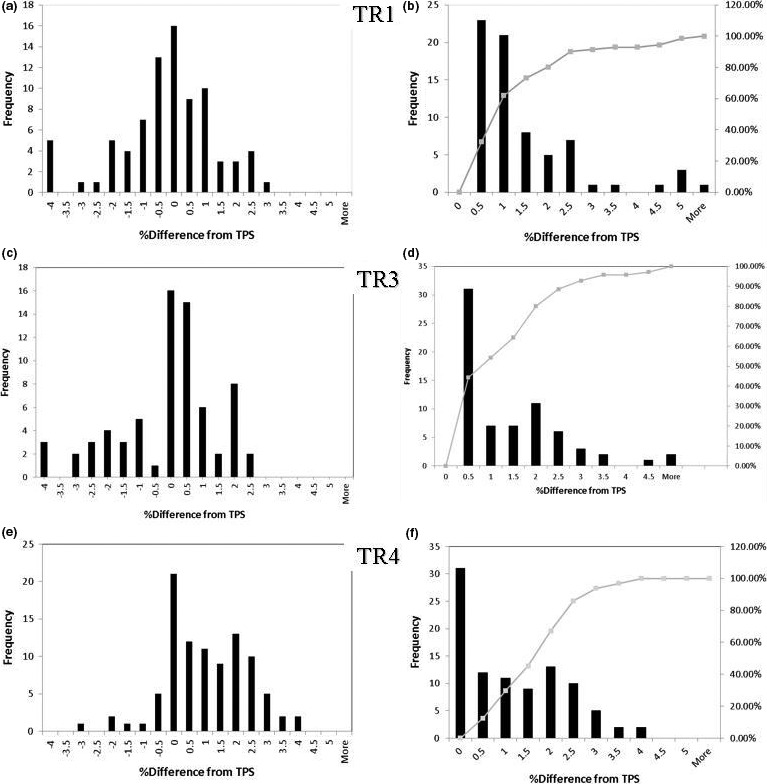
Distribution of percentage differences between test plans run on each gantry and the treatment planning system (TPS). Left panels show percentage differences between doses calculated by the TPS and absolute dose measurements. Right panels show cumulative distribution functions.

#### 3.C.2 Spot symmetry (Tolerance: ≤5%)

Table 4 shows symmetry between *σ*_*x*_ and *σ*_*y*_ for TR1, TR3, and TR4. The largest asymmetries are found for TR1, with a maximum of 4.09% at 245 MeV. For TR3 the maximum is 3.04% at 150 MeV, and for TR4 3.56% at 240 MeV.

**Table 4:** Symmetry: |(σ_x_‐σ_Y_)|/(σ_x_+σ_Y_)*100 (%) for MPTC gantries TR1, TR3, and TR4 for each energy between the spot *σ*s at a gantry angle of 0° at isocenter

Energy (MeV)	TR1	TR3	TR4
245	4.09	2.46	3.50
240	4.81	3.01	3.56
230	4.28	1.33	1.22
220	3.77	0.24	2.31
210	3.79	0.60	3.17
200	3.31	1.46	2.96
190	2.83	1.17	1.88
180	2.96	0.66	2.07
170	2.93	1.42	2.13
160	2.88	2.70	1.50
150	2.39	3.04	1.77
140	2.94	2.32	2.52
130	2.33	2.40	0.00
120	2.18	2.11	1.55
110	2.03	1.75	0.92
100	1.66	0.69	0.19
90	1.11	0.28	1.70
80	2.73	0.00	1.78
70	0.79	1.42	3.03

### 3.D MU linearity

Monitor unit linearity was found to be within tolerance (≤2% for >5 MU and ≤5% for ≤5 MU) for all gantries and for each energy delivered. Ratios were <1% for 70 MeV and increase as the energy increases. The minimum MU that can be delivered per spot at MPTC is 1 MU. This still gives an acceptable accuracy, because spots delivering the minimum MU are mostly a fraction of the overall number of spots in a treatment plan.

### 3.E Monitor end effect

Results for the end effect calculated with equation (1) are all within the 0.1% tolerance (≤3 MU here) given by TG142.^6^ Note that a negative value for the *x*‐intercept implies too much dose was delivered during start and termination of the beam, whereas a positive intercept implies underdosing caused by ramp‐up of the dose. This is opposite from the sign interpretations given by the method using equation (1).

The *x*‐axis intercepts of the lines fitted through the MU linearity data were all within tolerance for TR1, TR3, and TR4, with a maximum of –2.45 MU for 240 MeV.

### 3.F Dose rate dependence

Dose rate dependence was within 0.3% for all energies and dose rates tested for TR1, TR3, and TR4.

### 3.G Output vs gantry angle

Output variation with gantry angle was <0.3% for TR3 and TR4 for all energies measured. For TR1 it was larger than the tolerance of 1% for 240 MeV. The maximum was 1.6% for 240 MeV.

### 3.H Reproducibility and interrupted treatments

All doses were within 1 cGy of each other (which is 0.2% of the planned dose) for the same plans delivered repeatedly. The profiles agreed within 1%/1 mm for 100% of the points.

For the interrupted treatment the profiles agreed within 1%/1 mm for 96% of the points. Profiles across the interruption show a decrease in dose of 1.1%. This was repeated two times, and the average of the maximum dose difference was a decrease of 0.8%.

### 3.I Test plan comparison

The distribution of percentage differences between the point doses for the test plans calculated with the TPS and those measured are shown in Fig for TR1, TR3, and TR4. For TR1 90% of the planned and measured doses agreed within 2.5%. For TR3 and TR4 90% of the doses agreed within 2.75%.

The distribution of percentage differences between point doses for the test plans measured for TR4 and those for TR1 and TR3 show similar trends. For the TR4–TR1 comparison 90% of planned and measured doses agreed within 2.9%, and for the TR4–TR3 comparison 90% of the agreed within 2.75%.

The distribution of measurements was skewed higher than the calculated TPS doses for TR4 for a majority of the plans. For TR1, measurements were more evenly distributed across an average of 0%.

### 3.J Range shifters

The largest differences between the range shifter WETs measured with the Giraffe and the theoretical values for R90 were within 0.88%. This corresponds to a 0.02‐cm difference in the WET for the 2‐cm range shifter. The WETs used for the range shifters in the TPS were 5.7, 3.42, and 2.28 cm. The WET for the kVue One Proton couch base was measured as 7 mm. The Hounsfield units of the couch base obtained from a CT image were changed to represent the measured WET and inserted into each patient's CT image.

## 4 Conclusion

We found reasonable agreement between the TPS and measurements without using a double Gaussian function to model the spot profiles in the TPS as suggested by other authors.^1^, ^13^, ^14^, ^15^, ^16^ Although this was surprising, including a double Gaussian function in the TPS model did not markedly increase agreement between measured and calculated data from the TPS. The same beam model could be used to model all the MPTC gantries, and the machines were declared dosimetrically equivalent. We also showed agreement between spot profiles and IDDs measured at different sites for this system.

## Conflict Of Interest

Dr. Dong reports personal fees from Varian Medical Systems, outside the submitted work; Other authors have no conflicts of interest to report
